# Supplementary material for: Effect of Meropenem on Conjugative Plasmid Transfer in Klebsiella pneumoniae
Source: Int J Mol Sci. 2024 Dec 8;25(23):13193. doi: 10.3390/ijms252313193 (PMC11642842; doi:10.3390/ijms252313193)
Supplement: Supplementary file 1 [file ijms-25-13193-s001.zip › ijms-3334104-supplementary materials.pdf]

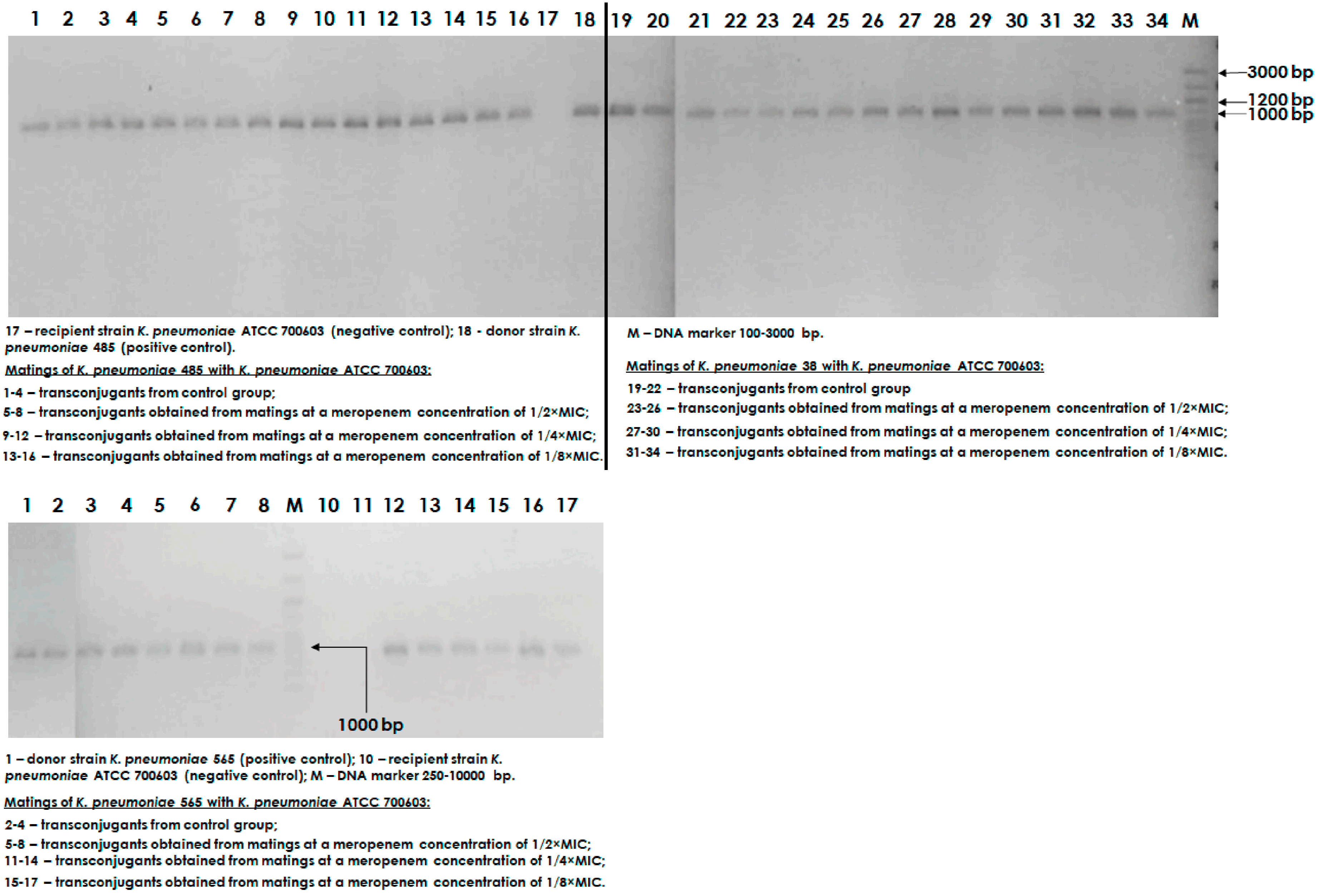

Figure S1. Agarose gel electrophoresis of *K. pneumoniae* ATCC 700603 transconjugants amplification products, 10V/cm, 0.9% agarose, DNA size marker 100-3000 bp or 250-10000 bp.

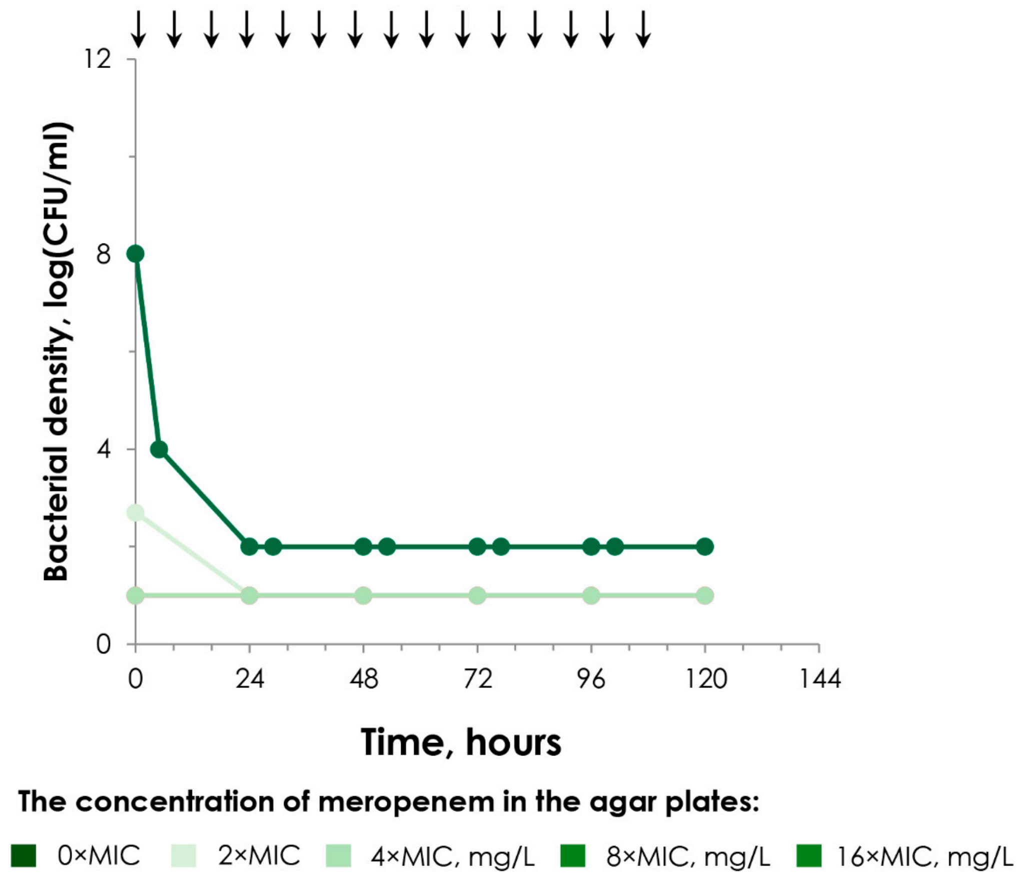

Figure S2. Time courses of the total bacterial population (0 × MIC) and meropenem-resistant (2×, 4×, 8× and 16 × MIC) sub-populations of recipient carbapenemase-non-producing strain *K. pneumoniae* ATCC 700603 (control) in pharmacodynamic experiments (high-dose meropenem exposure). Data are presented as arithmetic means (n=3). Arrows indicate the start of meropenem infusion.

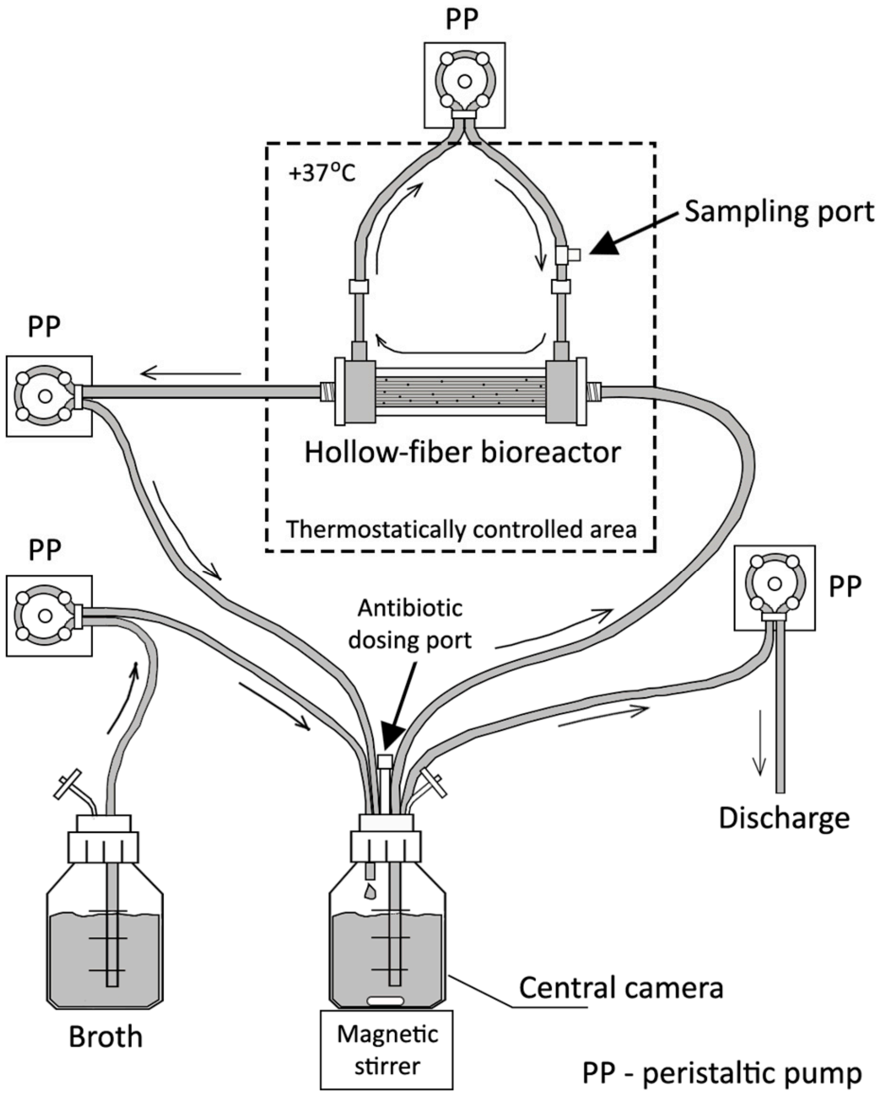

Figure S3. A schematic illustration of the hollow-fiber infection model (HFIM).
